# Extremely low frequency pulsed electromagnetic fields cause antioxidative defense mechanisms in human osteoblasts via induction of •O_2_^−^ and H_2_O_2_

**DOI:** 10.1038/s41598-017-14983-9

**Published:** 2017-11-06

**Authors:** Sabrina Ehnert, Anne-Kristin Fentz, Anna Schreiner, Johannes Birk, Benjamin Wilbrand, Patrick Ziegler, Marie K. Reumann, Hongbo Wang, Karsten Falldorf, Andreas K. Nussler

**Affiliations:** 1 Siegfried Weller Institute for Trauma Research, Eberhard-Karls-Universität Tübingen, Schnarrenbergstr. 95, D-72076 Tübingen, Germany; 2 Sachtleben GmbH, Hamburg, Spectrum UKE, Martinistraße 64, D-20251 Hamburg, Germany; 3 Wuhan Union Hospital, Tongji Medical College, Huazhong University of Science and Technology, Jiefang Dadao 1277#, 430022 Wuhan, China

## Abstract

Recently, we identified a specific extremely low-frequency pulsed electromagnetic field (ELF-PEMF) that supports human osteoblast (hOBs) function in an ERK1/2-dependent manner, suggesting reactive oxygen species (ROS) being key regulators in this process. Thus, this study aimed at investigating how ELF-PEMF exposure can modulate hOBs function via ROS. Our results show that single exposure to ELF-PEMF induced ROS production in hOBs, without reducing intracellular glutathione. Repetitive exposure (>3) to ELF-PEMF however reduced ROS-levels, suggesting alterations in the cells antioxidative stress response. The main ROS induced by ELF-PEMF were •O_2_^−^ and H_2_O_2_, therefore expression/activity of antioxidative enzymes related to these ROS were further investigated. ELF-PEMF exposure induced expression of *GPX3, SOD2, CAT* and *GSR* on mRNA, protein and enzyme activity level. Scavenging •O_2_^−^ and H_2_O_2_ diminished the ELF-PEMF effect on hOBs function (AP activity and mineralization). Challenging the hOBs with low amounts of H_2_O_2_ on the other hand improved hOBs function. In summary, our data show that ELF-PEMF treatment favors differentiation of hOBs by producing non-toxic amounts of ROS, which induces antioxidative defense mechanisms in these cells. Thus, ELF-PEMF treatment might represent an interesting adjunct to conventional therapy supporting bone formation under oxidative stress conditions, e.g. during fracture healing.

## Introduction

In 2000 osteoporosis caused approx. 9 million fractures worldwide^1^. Due to the anticipated demographic changes, this number is expected to be doubled by 2040^2,3^. Despite great surgical advances, osteoporotic fractures remain a major public health concern. Fracture healing is delayed and often accompanied by postoperative complications, which in turn negatively affects the general outcome^2,4^.

For almost 50 years various forms of electric and electromagnetic fields (EMFs) have been used to promote bone formation after fractures as well as for the treatment of osteoporosis^5^. Despite the overall positive effects of EMFs on osteotomies, spine fusions, as well as delayed and non-union fractures^6,7^, this technology remained a niche application, probably, because the modes of action are not sufficiently understood^8^. In human bone EMFs are assumed to induce mechanisms similar to mechanical load, when a strain gradient develops. Compensation of the resulting pressure gradients in the interstitial fluid causes flow-related shear stress and electrical potentials^9^. However, the underlying molecular mechanisms are not explained as easily. A variety of physical models and theories try to elucidate the influence of EMFs on molecules as well as on biological and chemical processes. These include, among others, alterations in ion flux and membrane potential, re-organization of the cytoskeleton, action of voltage-sensitive enzymes, regulation of gene expression via EMF-responsive sequences, and modifications in the cells oxidative state^8,10,11^.

Recently, we characterized the effect of ten defined (10 to 90.6 Hz) extremely low-frequency pulsed EMFs (ELF-PEMFs termed CIT programs), generated by a medical device (Somagen®, Sachtleben GmbH, Hamburg, Germany), on the function of human osteoblasts (hOBs) and osteoclasts. CIT program #16, with a frequency of 16 Hz, most effectively induced proliferation and differentiation of hOBs^12^. Our data is in line with other reports showing increased matrix production and mineralization in EMF treated cells^9,13–16^. Application of EMFs has been reported to activate transmembrane receptors, e.g. parathyroid hormone, insulin, transferrin or calcitonin receptors, thus initiating signaling cascades^6^. Our preceding experiments showed increased mitochondrial activity and activation of the ERK1/2 signaling cascade directly following ELF-PEMF exposure. ERK1/2 activation was crucial for the observed effects of ELF-PEMF on hOBs^12^. This observation is supported by the work of Yumoto, showing that EMF exposure induces osteogenic differentiation of MC3T3-E1 cells in an ERK1/2 and p38 dependent manner^17^. The work of Friedman links this observation to the cellular scavenging system by showing that radiofrequency EMF-dependent activation of ERK1/2 is mediated by reactive oxygen species (ROS) produced by NADPH-oxidases^18^. The level of EMF-induced ROS showed to be dependent on the field strength and frequency and may thus trigger diverse cellular responses, ranging from activation of signaling cascades (e.g. ERK1/2, JNK1-3 or p38) to oxidative stress induced cell death^11^. Exposure of cells to EMFs in the micro- and radio-frequency range seem to induce cellular stress, as could be seen by up-regulation of heat shock proteins or direct damage of the DNA^11^. However, far less is known about the cellular effect of ELF-PEMFs.

**Figure 1:**
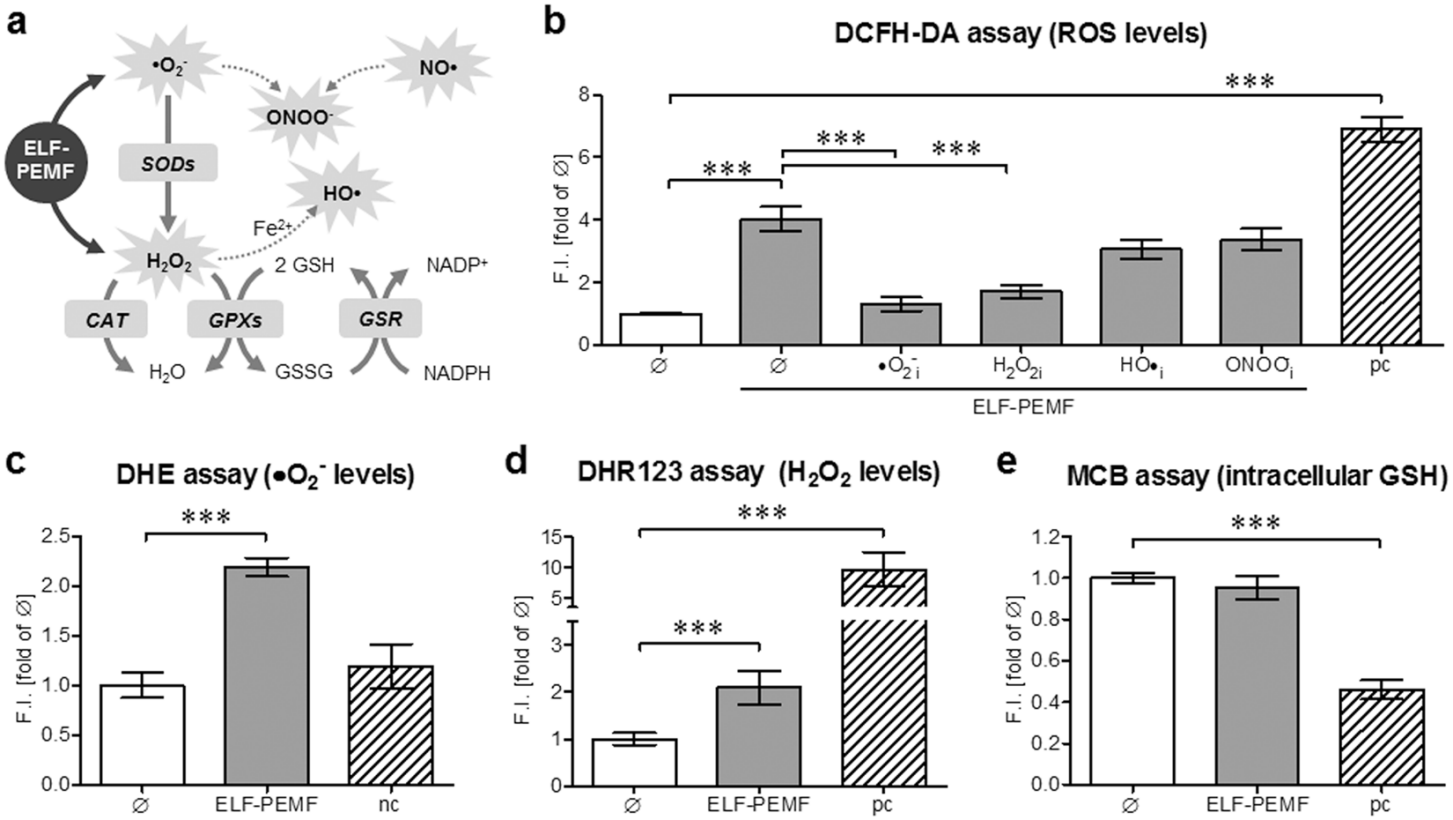
ELF-PEMF single exposure induces formation of •O_2_^−^ and H_2_O_2_ in hOBs. (**a**) Schematic overview on the investigated ROS cascade. Directly after single exposure (7 min) to ELF-PEMF intracellular ROS and GSH levels were determined in hOBs (N = 12; n = 4), using different fluorescent probes: (**b**) DCFH-DA assay was used to detect •O_2_^−^, H_2_O_2_, HO• and ONOO^−^. To trap the ROS hOBs were co-incubated with either 25 µM α-tocopherol (•O_2_^−^_i_), 10 mM sodium-pyruvate (H_2_O_2i_), 250 mM DMSO (HO•_i_) or 100 µM uric acid (ONOO^−^_i_); (**c**) DHE assay was used to detect •O_2_^−^; (**d**) DHR123 assay was used to detect H_2_O_2_ and (**e**) MCB assay was used to detect intracellular GSH. Results were normalized to unstimulated hOBs (Ø). hOBs stimulated with 0.01% H_2_O_2_ were used as positive control (pc) or negative control (nc) to show functionality and specificity of the respective assays. ***p < 0.001 as indicated (Kruskal-Wallis test followed by Dunn’s multiple comparison test, α = 0.05).

In our preceding experiments ELF-PEMF exposure induced mitochondrial activity in hOBs^12^. As a by-product of the mitochondrial respiratory chain ROS (superoxide anion (•O_2_^−^), hydrogen peroxide (H_2_O_2_), hydroxyl radicals (HO•) and peroxinitrite anion (ONOO^−^)) are produced. In low amounts these ROS trigger various cellular processes, e.g. activation of MAPKinases or cell migration. However, their accumulation (oxidative stress) may damage cellular macromolecules including proteins and DNA, eventually causing cell death. Therefore, the cells constantly fight against excessive ROS. Enzymes involved in the intracellular antioxidant defense include superoxide dismutases (SODs), catalase (CAT), glutathione peroxidases (GPXs) and glutathione-disulfide reductase (GSR). A balance between these enzymes’ activities and intracellular levels of antioxidants e.g. glutathione (GSH) are essential for the survival of organisms and their health^19^ (for overview see Fig. 1a).

Reduced bone mineral density might result from reduced bone formation by osteoblasts and/or increased bone resorption by osteoclasts. Both processes are tightly controlled by ROS^20^, which may activate osteoclasts while inhibiting osteoblasts^21^. Resulting fractures and their surgical reposition imply oxidative stress for the patients, as characterized by increased lipid peroxidation levels up to 3 weeks after fracture^22^. It is conceivable that therapeutic strategies reducing oxidative stress could reduce bone degradation by osteoclast while favoring bone formation by osteoblasts^21^. This way reduction of oxidative stress could improve fracture healing and thus reduce the postoperative complication rate.

In our preceding work, hOBs with impaired function (AP activity and matrix mineralization) profited most from the ELF-PEMF exposure. The induction of mitochondrial activity and necessity of ERK1/2 activation points towards ROS as critical regulators for this process^12^. Thus, aims of this project were: (i) to identify specific ROS formed in hOBs after ELF-PEMF (CIT #16) exposure; (ii) to characterize the effect of this ELF-PEMF on the oxidative stress response in these cells; (iii) to investigate the effects of the identified ROS on hOBs function.

## Results

### Single exposure to ELF-PEMF induces formation of •O_2_^−^ and H_2_O_2_ in hOBs

Our preceding study showed that repetitive exposure to ELF-PEMF induced mitochondrial activity and improved hOBs function in an ERK1/2-dependent manner, suggesting that ROS play a crucial role in this process^12^. Therefore, we investigated whether ELF-PEMF exposure induces formation of ROS in hOBs. Therefore, ROS formation and intracellular GSH content were measured in hOBs (N = 12; n = 4) immediately after ELF-PEMF exposure, using different fluorescent based assays.

The most commonly known but unspecific DCFH-DA (2′,7′-dichlorofluorescin diacetate) assay indicated that single exposure to ELF-PEMF, significantly (4.0-fold, *p* < 0.001, α = 0.05) induced DCF fluorescence (ROS levels) in hOBs reaching levels comparable to 0.01% H_2_O_2_ (H_2_O_2_ = positive control). The DCFH-DA assay detects •O_2_^−^, H_2_O_2_, HO• and ONOO^−^. Thus, we used several radical scavengers to identify the ROS species formed by ELF-PEMF exposure. Incubation with either 25 µM α-tocopherol or 10 mM sodium-pyruvate, known to trap •O_2_^−^ and H_2_O_2_, significantly reduced ROS levels in hOBs following ELF-PEMF exposure. Incubation with 250 mM DMSO and 100 µM uric acid, known to trap HO• and ONOO^−^, could not reduce ROS levels in ELF-PEMF exposed cells (Fig. 1b).

In order to verify the formation of •O_2_^−^ ELF-PEMF exposed hOBs were analyzed using the DHE (dihydroethidium) assay. Fluorescent signals representing •O_2_^−^ levels increased by 2.2-fold (*p* < 0.001, α = 0.05) following ELF-PEMF exposure. Cells stimulated with 0.01% H_2_O_2_ were used as negative control (assay specificity / Fig. 1c). Nuclear accumulation of the fluorescent signal was confirmed microscopically (supplementary figure 1a).

The DHR123 (dihydrorhodamine 123) assay was used to measure the formation of H_2_O_2_ in ELF-PEMF exposed hOBs. Fluorescent signal representing H_2_O_2_ levels increased by 2.1-fold (*p* < 0.001, α = 0.05) following ELF-PEMF exposure. Cells stimulated with 0.01% H_2_O_2_ (positive control) showed a 9.7-fold (*p* < 0.001, α = 0.05) increase in fluorescence (Fig. 1d). Fluorescent signals were confirmed microscopically (supplementary figure 1b).

Intracellular GSH content was detected using the monochlorobimane (MCB) assay. ELF-PEMF exposure did not affect intracellular GSH levels in hOBs. Solely cells stimulated with 0.01% H_2_O_2_ (positive control) showed a decrease (0.5-fold, *p* < 0.001, α = 0.05) in fluorescence/intracellular GSH (Fig. 1e).

### Single ELF-PEMF exposure induces ROS formation in hOBs comparable to 0.001% H_2_O_2_

**Figure 2:**
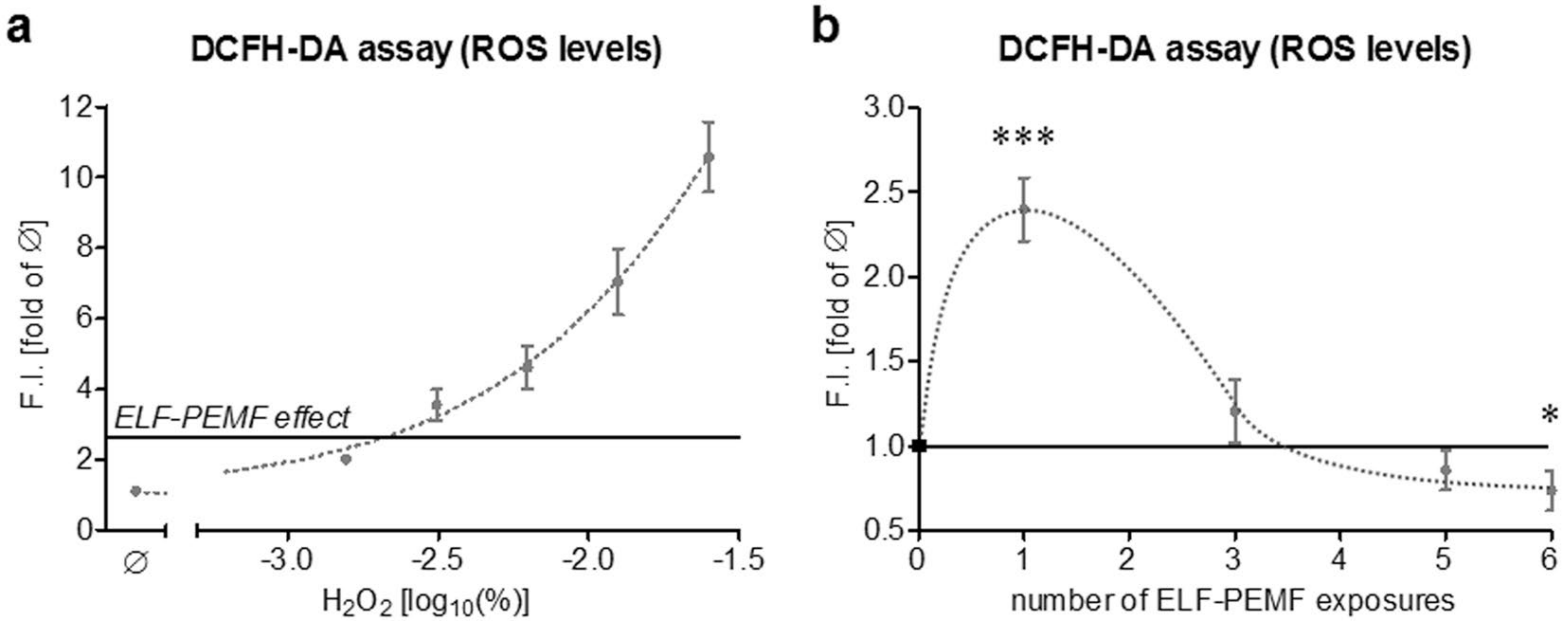
Effect of single and repetitive exposures to ELF-PEMF on ROS formation in hOBs. (**a**) Comparison of the ROS level in hOBs (N = 6, n = 4) after single exposure to ELF-PEMF (black line) and stimulation with different concentrations (0, 0.0015625, 0.003125, 0.00625, 0.0125 and 0.025%) of H_2_O_2_. (**b**) ROS levels in hOBs (N = 12, n = 4) after single and repetitive exposures to ELF-PEMF (7 min per day). ROS levels were determined using the DCFH-DA assay. Results are given as fold of unstimulated hOBs (Ø). *p < 0.05 and ***p < 0.001 as compared to corresponding unstimulated hOBs (two-tailed Mann-Whitney test, α = 0.05).

In order to estimate the amount of ROS produced by ELF-PEMF exposure, DCFH-DA assay was performed on hOBs (N = 6, n = 4) stimulated with different concentrations (0, 0.0015625, 0.003125, 0.00625, 0.0125 and 0.025%) of H_2_O_2_. DCF fluorescence (ROS levels) after single exposure to ELF-PEMF was comparable to stimulation with 0.001% H_2_O_2_ (Fig. 2a).

### Repetitive ELF-PEMF exposure reduces ROS formation in hOBs

During the osteogenic differentiation process hOBs (N = 12; n = 4) were exposed to ELF-PEMF 5 times per week (Monday to Friday), for 7 min each day. On day 0, 2, 4 and 7 ROS levels were determined immediately after ELF-PEMF exposure using the DCFH-DA assay.

As described before single exposure to ELF-PEMF caused a significant increase in DCF fluorescence (ROS levels)in hOBs. After the third exposure (day 2) DCF fluorescence (ROS levels) was comparable to unstimulated cells. After 5 and 6 exposures (days 4 and 7) DCF fluorescence (ROS levels) was even decreased (−14.5% and −26.5% respectively) compared to untreated hOBs (Fig. 2b). These data suggest that the initial small increase in ROS levels following ELF-PEMF exposure does not harm hOBs but stimulates their intracellular oxidative stress defense.

### ELF-PEMF exposure induces expression of genes involved in oxidative stress defense

**Figure 3:**
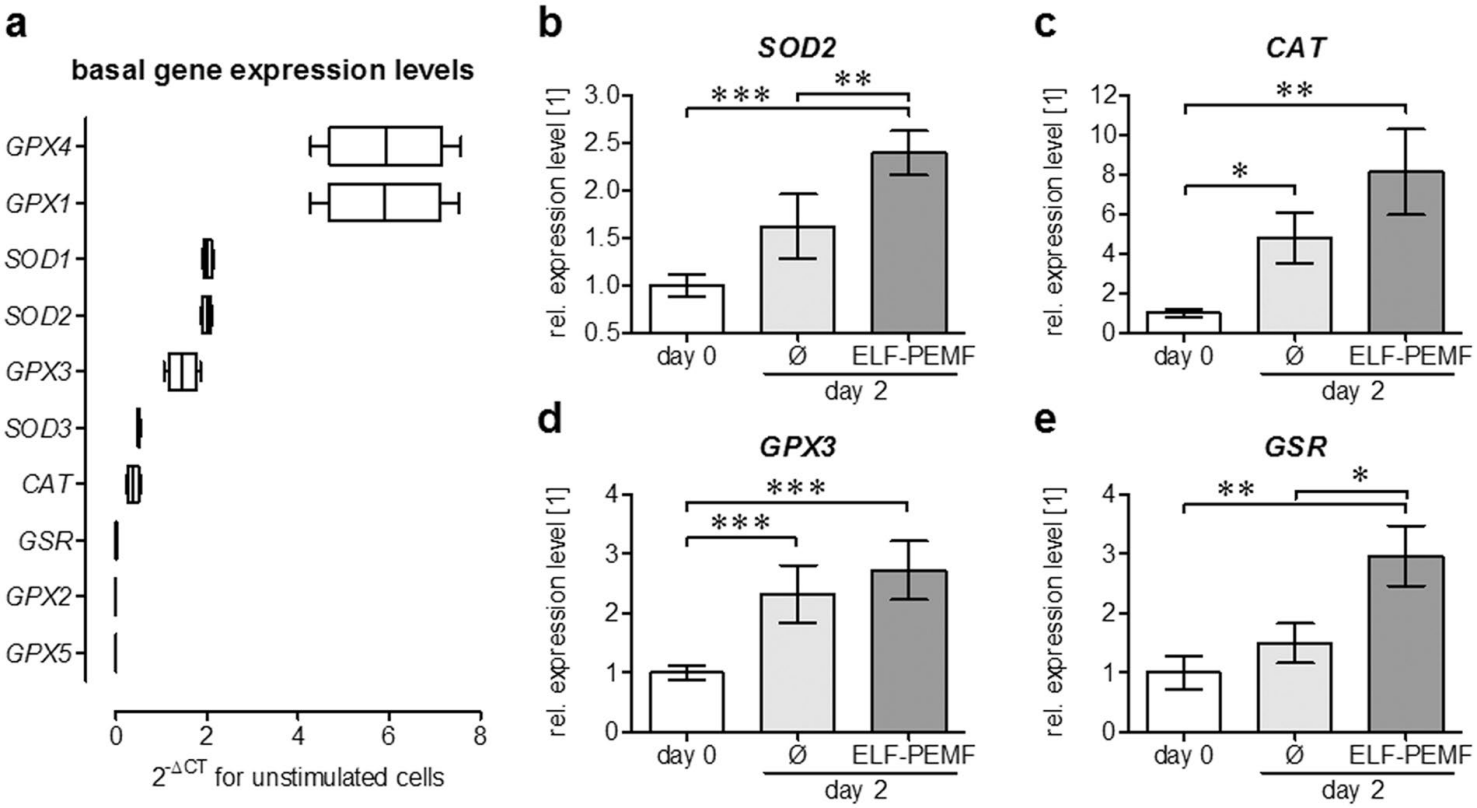
ELF-PEMF exposure induces gene expression of intracellular antioxidative enzymes in hOBs. (**a**) Basal expression levels of oxidative stress (•O_2_^−^ and H_2_O_2_) related genes in hOBs was determined using the RT² Profiler PCR Array human oxidative stress plus (Qiagen, Hilden, Germany). (**b**–**e**) Semi-quantitative RT-PCR revealed that expression of *SOD2, CAT, GPX3* and *GSR* was increased by repetitive exposures to ELF-PEMF (7 min per day) during the osteogenic differentiation of hOBs. Densitometric analysis (ImageJ software) was performed with individual samples (N = 12) analyzed twice (n = 2) to reduce small loading differences. The mean signal intensity of cells on day 0 was set as reference. *p < 0.05, **p < 0.01 and ***p < 0.001 as indicated (Kruskal-Wallis test followed by Dunn’s multiple comparison test, α = 0.05).

Enzymes involved in the intracellular degradation of •O_2_^−^ and H_2_O_2_ include superoxide dismutases (SODs), catalase (CAT), glutathione peroxidases (GPXs) and glutathione-s-reductase (GSR). We first screened the basal expression levels of the different isoforms using the real-time RT-PCR based RT² Profiler PCR Array human oxidative stress plus using a cDNA pool of all samples. Highest expression levels showed *GPX1* and *GPX4*, followed by *SOD1*, *SOD2* and *GPX3*. Basal expression levels of *SOD3* and *CAT* were lower. Expression of *GSR*, *GPX2* and *GPX5* was close to the detection limit (Fig. 3a).

To investigate the ELF-PEMF effect on the intracellular oxidative stress defense hOBs (N = 12; n = 2) were exposed to ELF-PEMF for 7 min each day during the osteogenic differentiation process. After 2 days (3 exposures), when ROS levels reduced, gene expression was again screened with the pooled samples (RT² Profiler PCR Array human oxidative stress plus). Results were confirmed with conventional RT-PCR using the individual samples. Expression of *GPX1*, *GPX4* and *SOD1* remained unchanged. *SOD2* expression rose (1.6-fold) only slightly during the osteogenic differentiation process. Exposure to ELF-PEMF, however, increased *SOD2* expression (2.4-fold, *p* < 0.001, α = 0.05 / Fig. 3b). Although *CAT* expression increased by the osteogenic differentiation process (4.8-fold, *p* = 0.018, α = 0.05), ELF-PEMF exposure was able to further increase *CAT* expression levels (8.1-fold, *p* = 0.0013, α = 0.05 / Fig. 3c). *GPX3* was significantly increased both by osteogenic differentiation (2.3-fold, *p* < 0.001, α = 0.05) and ELF-PEMF exposure (2.7-fold, *p* < 0.001, α = 0.05 / Fig. 3d). *GSR* expression levels only increased by ELF-PEMF exposure (3.0-fold, *p* = 0.0027, α = 0.05 / Fig. 3e).

### ELF-PEMF induces protein levels and function of antioxidant enzymes in hOBs

**Figure 4:**
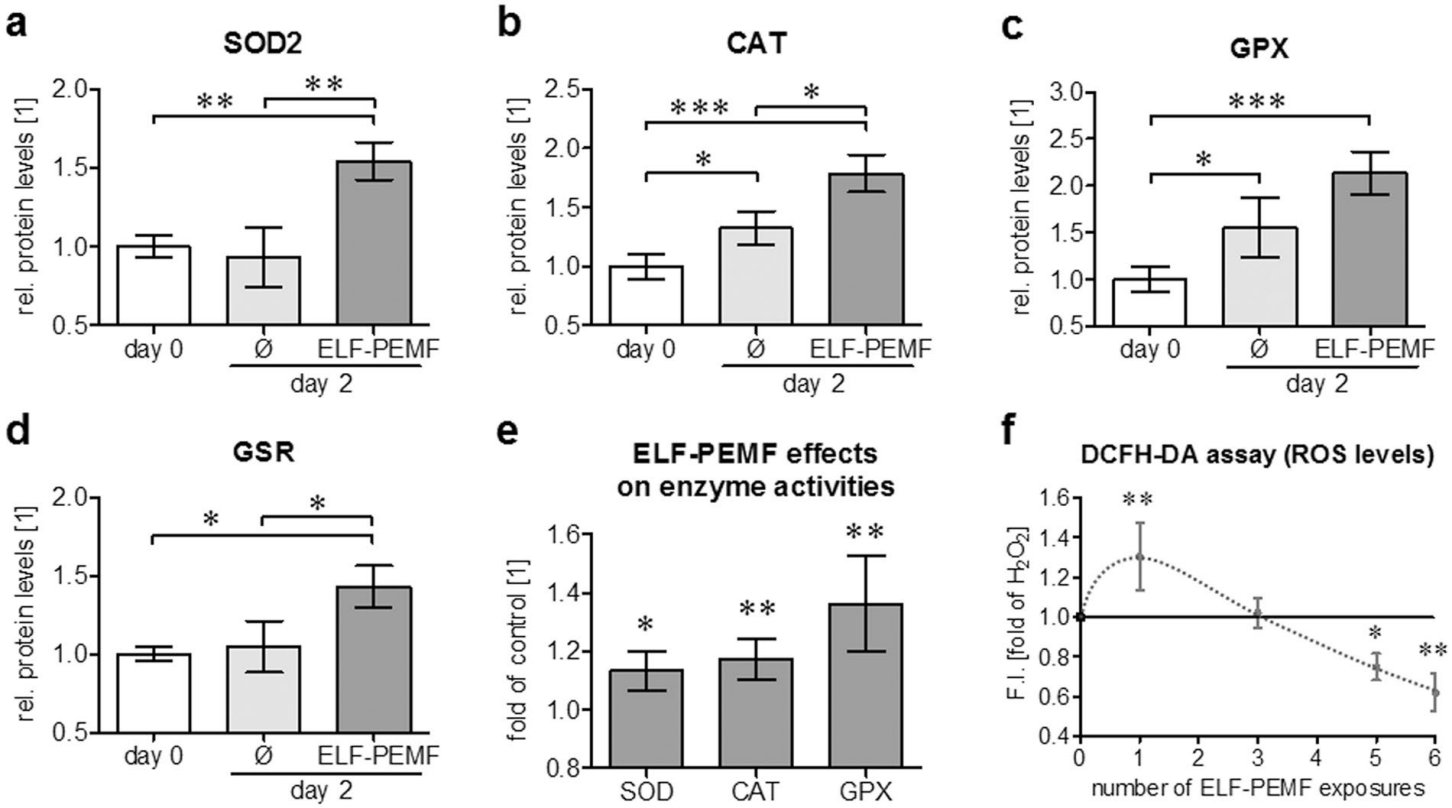
ELF-PEMF exposure increases protein levels and function of antioxidative enzymes in hOBs. hOBs were osteogenically differentiated with or without daily exposure to ELF-PEMF. After 2 days protein levels of SOD2, CAT, GPXs and GSR were determined by Western blot. (**a**–**d**) Signal intensities were quantified by densitometric analysis using the ImageJ software. Individual samples (N = 9) were analyzed twice (n = 2) to reduce small loading differences. The mean signal intensity of cells on day 0 was set as reference. *p < 0.05, **p < 0.01 and ***p < 0.001 as indicated (Kruskal-Wallis test followed by Dunn’s multiple comparison test, α = 0.05). (**e**) Changes in enzyme activities (N = 10; n ≥ 2) were determined using test kits. (**f**) ROS levels in hOBs (N = 12, n = 4) after single and repetitive exposures to ELF-PEMF (7 min per day) and additional H_2_O_2_ (0.01%) stimulation. ROS levels were determined using the DCFH-DA assay. Results are given as fold of H_2_O_2_ treated hOBs. *p < 0.05 and **p < 0.01 as compared to unexposed cells on day 2 (two-tailed Mann-Whitney test, α = 0.05).

In order to verify the observed gene expression changes on the protein levels hOBs (N = 9) were osteogenically differentiated with and without daily exposure to ELF-PEMF. After 2 days protein levels of SOD2, CAT, GPXs and GSR were determined by Western blot (representative blots in supplementary figure 3). Densitometric analysis revealed that SOD2 protein level was only induced by ELF-PEMF exposure (1.5-fold, *p* = 0.0017, α = 0.05) and not by osteogenic differentiation itself (Fig. 4a). Similar to the gene expression levels, CAT protein levels were increased already during the osteogenic differentiation process (1.3-fold, *p* = 0.0279, α = 0.05) but were further induced by ELF-PEMF exposure (1.8-fold, *p* < 0.001, α = 0.05 / Fig. 4b). Comparable to CAT, GPX protein levels were increased by the osteogenic differentiation process (1.6-fold, *p* = 0.0247, α = 0.05) and further induced by ELF-PEMF treatment (2.2-fold, *p* < 0.001, α = 0.05 / Fig. 4c). GSR protein levels only increased by ELF-PEMF exposure (1.4-fold, *p* = 0.021, α = 0.05 / Fig. 4d).

In order to investigate whether the observed increases in SOD, CAT and GPX protein levels also correlate to increased enzyme activities we measured enzyme activities of SOD, CAT and GPX in protein lysates from the same hOBs (on day 2 with and without ELF-PEMF exposure) using commercially available test kits. As compared to unexposed hOBs, ELF-PEMF exposure induced the measured enzyme activities by 11.3%, 11.7% and 13.6%, respectively (Fig. 4e).

### Repetitive exposure to ELF-PEMF effectively reduces ROS in hOBs

As described before hOBs (N = 12; n = 4) were exposed to ELF-PEMF 5 times per week (Monday to Friday), for 7 min each day, during the osteogenic differentiation process. On day 0, 2, 4 and 7 ROS levels were determined immediately after ELF-PEMF exposure using the DCFH-DA assay with additional H_2_O_2_ stimulation (0.01%). Single exposure to ELF-PEMF further increased DCF fluorescence (ROS levels) in hOBs treated with H_2_O_2_. After the third exposure (day 2) DCF fluorescence (ROS levels) was comparable to H_2_O_2_ treated cells. After 5 and 6 exposures (days 4 and 7) DCF fluorescence (ROS levels) was even decreased (−25.1% and −38.1% respectively) compared to H_2_O_2_ treated cells (Fig. 4f). These data suggest that the observed increase in antioxidative enzymes in ELF-PEMF exposed OBs effectively reduces ROS in these cells.

### ROS induction is necessary to improve the osteogenic function of hOBs by ELF-PEMF

In order to investigate whether these small increases in ROS are necessary for the observed positive effect of ELF-PEMF exposure in hOBs function, hOBs (N = 12, n = 4) were osteogenically differentiated with or without daily exposure to ELF-PEMF. Additionally cells were stimulated with 0.001% H_2_O_2_. To trap the ROS ELF-PEMF exposed hOBs were co-incubated with either 25 µM α-tocopherol (•O_2_^−^_i_) or 10 mM sodium-pyruvate (H_2_O_2i_).

**Figure 5:**
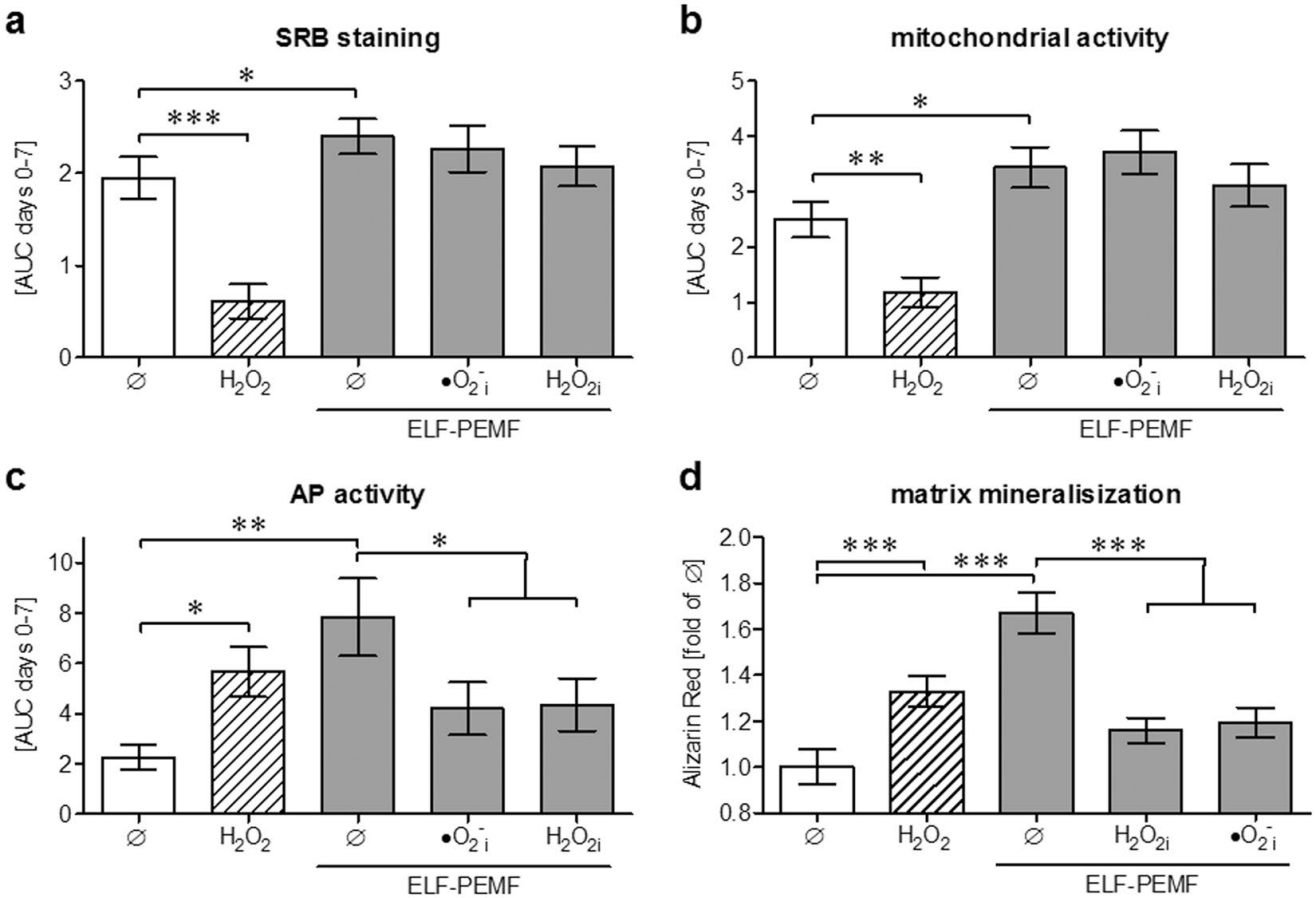
ROS induction is necessary to improve the osteogenic function of hOBs by ELF-PEMF. HOBs (N = 12, n = 4) were osteogenically differentiated with or without daily exposure to ELF-PEMF. To simulate oxidative stress cells were stimulated with 0.001% H_2_O_2_ (shaded bars). To trap the ROS ELF-PEMF exposed hOBs were co-incubated with either 25 µM α-tocopherol (•O_2_^−^_i_) or 10 mM sodium-pyruvate (H_2_O_2i_). (**a**–**c**) On days 0, 2, 4 and 7 total protein content (SRB staining), mitochondrial activity (Resazurin conversion) and AP activity were measured. Results are given as AUC (area under the curve) from day 0 to day 7. (**d**) After 14 days the formed mineralized matrix was quantified by Alizarin Red staining. Data are given as fold of unstimulated cells. *p < 0.05, **p < 0.01 and ***p < 0.001 as indicated (Kruskal-Wallis test followed by Dunn’s multiple comparison test, α = 0.05).

Within the first week of differentiation total protein content and mitochondrial activity decreased when cells were stimulated with 0.001% H_2_O_2_, indicating towards reduced cell numbers. On the contrary ELF-PEMF exposure slightly increased total protein content and mitochondrial activity. Co-incubation with the radical scavengers did not alter total protein content and mitochondrial activity (Fig. 5a & b).

In the same time frame the cells AP activity (normalized to total protein content) increased both by 0.001% H_2_O_2_ (2.5-fold, *p* = 0.0267, α = 0.05) and ELF-PEMF (3.5-fold, *p* = 0.002, α = 0.05) treatment. However, co-incubation with the radical scavengers significantly reduced the cells AP activity despite exposure to ELF-PEMF (−46.5% and −54.1% / Fig. 5c).

After 14 days the formed mineralized matrix was quantified by Alizarin Red staining. Similar to the AP activity more mineralized matrix was formed by hOBs treated with 0.001% H_2_O_2_ (1.3-fold, *p* < 0.001, α = 0.05) and ELF-PEMF (1.7-fold, *p* < 0.001, α = 0.05). Co-incubation with the radical scavengers effectively prevented formation of mineralized matrix despite exposure to ELF-PEMF (Fig. 5d).

## Discussion

In our previous work we could identify a specific ELF-PEMF (16 Hz)which favors proliferation and osteogenic differentiation of hOBs with reduced baseline activities. This effect was accompanied by increased mitochondrial activity and activation of the ERK1/2 signaling cascade^12^. Similar to JNK and p38, ERK1/2 is reported to be directly affected by oxidative stress stimuli^23,24^, often produced as a by-product of the mitochondrial respiratory of the mitochondrial respiratory chain. As a consequence we investigated the effect of this specific ELF-PEMF on ROS formation in hOBs. Single exposure to this ELF-PEMF induced formation of mainly •O_2_^−^ and H_2_O_2_ in hOBs. This is in line with the work of Friedman^18^, showing increased ROS formation resulting in activation of MAPKinases following EMF exposure at higher energetic mobile phone frequencies. This work postulates activation of NADPH-oxidases being responsible for the accumulation of ROS. However, in our system, ELF-PEMF did not affect expression of NADPH-oxidases and the correlated DUOX1 and 2 (data not shown). Thus, further investigations need to be done to identify the actual source of the generated •O_2_^−^ and H_2_O_2_. The work of Storch and colleagues suggests that lower frequency EMFs might cause alterations in glycolysis and TCA cycle in cancer cells^25^.

Repetitive ELF-PEMF exposures no longer induced ROS in hOBs. Interestingly more than 3 ELF-PEMF exposures even reduced ROS levels. These data point towards an adaptive mechanism in response to the initial increase in ROS. This hypothesis is supported by the work of Calcabrini, showing that ELF-EMFs may induce slight oxidative stress that does not harm the cells’ metabolic capacity or viability^26^. In this work single exposure to ELF-EMFs could not increase expression of antioxidative enzymes, supporting our hypothesis that the expression of antioxidative enzymes is based on an adaptive mechanism. Blank and Goodman on the other hand identified specific EMF responsive DNA sequences (nCTCTn sequences), which may directly regulate expression of genes, e.g. *c-myc* or *hsp70*, by EMF within a short time frame^27,28^. Checking the 5’-flanking sequence (1000 bp length / promoter) of our ELF-PEMF regulated genes we could not find nCTCTn-sequences, being aware, that this does not exclude the presence of other EMF responsive DNA sequences in these promoter regions.

In our case however, adaption to the ROS formed took several days. Consequently, we screened expression levels of genes associated with intracellular oxidative stress responses after 2 days when an effect of ROS levels was measurable. With •O_2_^−^ and H_2_O_2_ being the main ROS formed after ELF-PEMF exposure in our cells, we focused on the expression and activity of superoxide dismutases (SODs), catalase (CAT), glutathione peroxidases (GPXs) and glutathione-disulfide reductase (GSR) being the main enzymes involved in their mitochondrial degradation. Basal gene expression levels were highest for *GPX1* and *GPX4*, followed by *SOD1*, *SOD2*, *GPX3*, *SOD3* and *CAT*. Expression of *GSR*, *GPX2* and *GPX5* was close to the detection limit. The gene expression array indicates that solely expression of *GPX3, SOD2, CAT* and *GSR* raised during the osteogenic differentiation process which further increased by ELF-PEMF treatment. These results were confirmed by conventional RT-PCR and Western blot. Furthermore, enzyme activity of GPX, SOD and CAT were significantly increased after repetitive ELF-PEMF exposures. The increase in antioxidative enzyme activity effectively reduced ROS levels, when hOBs were additionally challenged with H_2_O_2_, pointing towards an overall protective effect of the repetitive short-term ELF-PEMF exposure.

Our data is supported by the work of Di Carlo, reporting that mechanical and EMF stimulation protected chicken embryos from oxidative stress. However, the observed effect was strongly dependent on the duration of the exposure^29^. Sun and colleagues, who exposed mice for 4 h daily over a period of 4 weeks, reported adverse effects. In their model ELF-PEMF exposure induced oxidative stress along with reduced activity of CAT, GPX, SOD2 and GSR^30,31^. Similarly, the work of Jiang showed damaging effects of long term PEMF exposure in rat brain being mediated by oxidative stress^32^. On the other hand, the work of Buldak shows increased SOD and GPX activity in AT478 murine squamous carcinoma cells following short-term exposure to 50 Hz ELF-EMF^33^. Similarly, Emre and colleagues reported an increased SOD activity in liver tissue of rat exposed to ELF-EMFs with varying frequency and pulse number^34^. Falone and colleagues also reported an increase in SOD activity in human neuroblastoma cells following PEMF exposure^35^. These data show that the observed effects strongly depend on the exposure time as well as the field strength and frequency of the applied EMF.

In our experiments exposure to ELF-PEMF was performed once per day (Monday to Friday) for 7 min. Repetitive exposure to ELF-PEMF induced expression and activity of antioxidant enzymes presumably as a response to the formed ROS. This is supported by the data of Raggi and colleagues showing that repetitive exposure (27 min per day for 10 days) to ELF-EMF reduced oxidative stress measures in blood of healthy volunteers. In this study oxidative stress levels normalized within 1 month after the treatment^36^.

Regarding bone health, Lei and colleagues could show that repetitive exposure to EMFs could support bone formation in ovariectomized (osteoporotic) mice. The Effect however was strongly dependent on the frequency range applied, while lower frequencies induced osteoblast function higher frequencies inhibit osteoclast function. Thus a broad band EMF, applied 3 h daily over a period of 8 weeks, had the strongest effect on the bone structure in their model^37^. This is in line with our previous work, showing that the ELF-PEMF most effectively inducing osteoblast function did not affect osteoclast function^12^.

Both osteoblasts and osteoclast function are reported to be regulated by ROS, while increased ROS production favors osteoclasts^20,21^. This is in line with the reports showing that reduction in antioxidant enzyme activities^38^ and increased oxidative stress being associated with osteoporosis^21^. This could explain the studies showing a detrimental effect of ROS formed by higher energetic EMFs.

However, our data clearly show that small initial activation of ROS is essential to promote the cells’ osteogenic differentiation, which is in line with the work of Lee and colleagues^39^. A possible explanation is given by the work of Chen and colleagues showing that the osteogenic differentiation of mesenchymal stem cells is strongly dependent on a coordinated regulation of mitochondrial biogenesis and the antioxidant enzymes SOD and CAT^40^. In our model, scavenging the formed •O_2_^−^ and H_2_O_2_ in the ELF-PEMF treated cells abrogated its positive effect on hOBs function (AP activity and matrix mineralization), suggesting that the formed ROS do not harm the cells but initiate the cells antioxidative response.

Considering that in our experiments repetitive short-term exposures to ELF-PEMF effectively decreased ROS levels by upregulation of antioxidant enzymes, ELF-PEMF exposure might represent an interesting adjunct to conventional therapy supporting bone formation under oxidative stress conditions, e.g. during fracture healing.

## Materials and Methods

### Ethics statement and patient information

All human studies were performed in accordance with the 1964 Declaration of Helsinki. HOBs isolation and all following experiments were in accordance with the ethical vote (387/2012BO) approved by the ethics committee of the medical faculty of the Eberhard-Karls-Universität and University clinic Tübingen. In accordance with the ethical vote informed consent (signature) was obtained from each patient donating bone samples for the isolation of hOBs used in the *in vitro* experiments. The donors’ average age was 69.3 ± 8.5 a (7 male and 17 female). Potential tumor patients or patients with viral or bacterial infections were excluded from this study.

### Isolation and expansion of hOBs

Briefly, cancellous bone was disintegrated mechanically and washed 3–5 times with PBS to remove residual blood. After 1 h of collagenase digestion (0.07% Collagenase II in PBS/Serva, Heidelberg, Germany) at 37 °C, cancellous bone was washed with PBS and released hOBs were transferred to cell culture flasks in culture medium (MEM/Ham’s F12, 10% FCS, 50 µM L-ascorbate-2-phosphate, 50 µM β-glycerol-phosphate) for expansion. Medium was changed every 4–5 days. Experiments were performed in passage 3 and 4 with cells seeded at a density of 20,000 cells/cm^2^ in cell culture medium. After 3 days of cell adherence cell culture medium was replaced by osteogenic differentiation medium (MEM/Ham’s F12, 1% FCS, 2 mM L-glutamine, 200 µM L-ascorbate-2-phosphate, 10 mM β-glycerol-phosphate, 25 mM HEPES, 1.5 mM CaCl_2_, 100 nM dexamethasone)^12^. Medium was changed every 3–4 days.

### Electromagnetic field application with the Somagen^®^ device

The ELF-PEMF was generated by the Somagen^®^ (Sachtleben GmbH, Hamburg, Germany), a medical device certified according to European law (CE 0482, compliant with EN ISO 13485:2012 + AC:2012) as previously described^12^. The specific ELF-PEMF applied here has a fundamental frequency of 16 Hz and an intensity of 6–282 µT (B field amplitude 6 mm above the applicator), which is emitted as groups of pulses (bursts) in sending-pause intervals. ELF-PEMF exposure was performed for 7 min each working day, i.e. Monday to Friday.

### Determination of ROS levels

To measure formation of ROS, different fluorescent probes were used^41^:

(i) for the most unspecific 2′,7′-dichlorofluorescein-diacetate (DCFH-DA) assay hOBs were incubated with 10 µM DCFH-DA for 25 min at 37 °C. Directly after ELF-PEMF exposure hOBs were washed twice with PBS. As positive control hOBs were stimulated with 0.001% H_2_O_2_. After 0, 5, 10 and 15 min the increase in fluorescence (ex/em = 485/520 nm) was detected by a plate reader, representing levels of •O_2_^−^, H_2_O_2_, HO• and ONOO^−^^42^. To trap the ROS hOBs were co-incubated with either 25 µM α-tocopherol (•O_2_^−^_i_), 10 mM sodium-pyruvate (H_2_O_2i_), 250 mM DMSO (HO•_i_) or 100 µM uric acid (ONOO^−^_i_)^41^.
(ii) to determine •O_2_^−^ levels hOBs were incubated with 10 µM dihydroethidium (DHE) for 25 min at 37 °C. After ELF-PEMF exposure hOBs were washed twice with PBS. As negative control (assay specificity) hOBs were stimulated with 0.001% H_2_O_2_. After 0, 5, 10 and 15 min the increase in fluorescence (ex/em = 544/590 nm) was detected by a plate reader. Cellular localization of the fluorescence was confirmed by fluorescence microscopy.
(iii) to measure H_2_O_2_ levels hOBs were incubated with 25 µM dihydrorhodamine (DHR123) for 25 min at 37 °C. After ELF-PEMF exposure hOBs were washed twice with PBS. As positive control hOBs were treated with 0.001% H_2_O_2_. After 0, 5, 10 and 15 min the increase in fluorescence (ex/em = 485/520 nm) was detected. Fluorescence signal was confirmed microscopically.

### Determination of intracellular GSH levels

To determine intracellular GSH levels hOBs were incubated with 10 µM monochlorobimane (MCB) for 25 min at 37 °C. Directly after ELF-PEMF exposure hOBs were washed twice with PBS. As assay control (decrease of intracellular GSH) hOBs were stimulated with 0.001% H_2_O_2_. After 0, 5, 10 and 15 min the decrease in fluorescence (ex/em = 355/460 nm) was measured with a plate reader^41^.

### Gene expression analysis

Total RNA was isolated using the Trifast reagent (Peqlab, Erlangen, Germany). Screening for expression of oxidative stress related genes was performed using the RT² Profiler PCR Array human oxidative stress plus (Qiagen, Hilden, Germany), with 16 RNA samples in 2 pools (each N = 8 donors) to minimize donor dependent variations. RNA purification, cDNA synthesis and the array itself were performed as indicated by the manufacturer using the advised products from Qiagen. Gene expression changes were confirmed for the individual samples by semi-quantitative RT-PCR using the KAPA2G Fast Ready Mix from Peqlab. Primer sequences and PCR conditions are summarized in Table 1. PCR products were separated by agarose gel electrophoresis and visualized by ethidium bromide (geldoc, INTAS, Göttingen, Germany). Individual samples were run twice (n = 2) to reduce variations caused by small loading differences. Signal intensities were quantified using the ImageJ software (NIH, Bethesda, USA). After background correction, signal intensities were normalized to the mean signal intensity of cells on day 0.

**Table 1:** Primer sequences and PCR conditions.

Gene	GeneBank ID [NM_]	Forward Primer	Reverse Primer	T_a_ [°C]	Amplicon [bp]
*CAT*	001752	ACCCTCGTGGGTTTGCAGTGA	CGAGCACGGTAGGGACAGTTCA	58	763
*GPX1*	000581.2	TGGGCATCAGGAGAACGCCA	GGGGTCGGTCATAAGCGCGG	60	199
*GPX3*	002084	CTGACGGGCCAGTACATTGA	TCCACCTGGTCGGACATACT	58	156
*GSR*	000637	AGGAGCTGGAGAACGCTGGC	CAATGGCCCAGAGCAGGCA	60	162
*SOD2*	001024465.1	GCAGCTGCACCACAGCAAGC	CGTGCTCCCACACATCAATCCCC	62	422
*GAPDH*	002046.4	GTCAGTGGTGGACCTGACCT	AGGGGTCTACATGGCAACTG	56	420

### Western Blot analysis

HOBs were lysed in freshly prepared ice-cold RIPA buffer. 35 µg total protein, quantified by micro Lowry, was separated by SDS page and transferred to nitrocellulose membranes. Membranes were blocked with 5% BSA in TBS-T for 1 h at ambient temperature followed by overnight incubation at + 4 °C with primary antibodies (sc-32886, sc-30147, sc-50508 and sc-30080 Santa Cruz Biotechnology, Heidelberg, Germany) diluted 1:1,000 in TBS-T. The next day membranes were incubated with the corresponding peroxidase-labeled secondary antibodies (1:10,000 in TBS-T) for 2 h at ambient temperature. GAPDH was used as a loading control. For signal development membranes were incubated for 1 min with ECL substrate solution. Chemiluminescent signals, detected by a CCD camera (INTAS, Göttingen, Germany), were quantified using the ImageJ software.

### Enzyme activity assays

Activities of SOD2, GPX and CAT were measured in cell lysates using commercially available test kits (Sigma Aldrich, Munich, Germany). Enzyme activities were normalized to total protein content determined by micro Lowry. Results were normalized to corresponding untreated cells.

### Sulforhodamine B (SRB) staining to assess total protein content

Total protein content was determined by SRB staining of ethanol fixed (1 h at −20 °C) cells. Cells were stained with 0.4% SRB (in 1% acetic acid) for 20 min at ambient temperature. Cells were washed 4–5 times with 1% acetic acid to remove unbound SRB. Bound SRB was resolved by with 10 mM unbuffered TRIS solution (pH ~ 10.5). Resulting absorption (λ = 565 nm) was determined with a plate reader^43^.

### Resazurin conversion assay to assess mitochondrial activity

For measuring mitochondrial activity 1/10 volume of a 0.025% (w/v) resazurin solution (in DPBS) was added to the cells. After 30 min of incubation at 37 °C, fluorescence intensity of the formed resorufin was measured (ex/em = 540/590 nm) and corrected to background control (solvent mixture without cells). Fluorescent intensities were normalized to the level of day 0 control^12^.

### AP activity measurement

For measuring AP activity hOBs were incubated with reaction buffer (0.2% p-nitrophenyl-phosphate, 50 mM glycine, 1 mM MgCl_2_, 100 mM TRIS, pH 10.5) at 37 °C. Resulting formation of p-nitrophenol (pNP) was determined photometrically (λ = 405 nm) and corrected to background control (solvent mixture without cells). Signals were normalized to relative cell numbers determined by SRB staining^12^.

### Assessing matrix mineralization by Alizarin Red staining

HOBs were fixed with ice cold ethanol for 1 h. After 3 times washing with tap water, cells were incubated with 0.5% Alizarin Red solution (pH 4.0) for 30 min at ambient temperature followed by 3 additional washing steps. Bound Alizarin Red staining was resolved with 10% Cetylpyridiumchloride solution and quantified photometrically (λ = 562 nm)^12^.

### Statistics

Results are expressed as bar diagrams (mean ± 95% CI). The precise number of biological (N) and technical (n) replicates for each experiment is given in the figure legends. Data sets were compared by two-tailed Mann-Whitney test (2 groups) or non-parametric Kruskal Wallis test followed by Dunn’s multiple comparison test (multiple groups/GraphPad Prism Software, El Camino Real, USA). *p* < 0.05 at an α = 0.05 was taken as minimum level of significance.

### Data availability

The datasets generated during and analyzed during the current study are available from the corresponding author on reasonable request.

## Electronic supplementary material

Supplementary Figure 1

Supplementary Figure 2

Supplementary Figure 3
