# Supplementary material for: Extremely low frequency pulsed electromagnetic fields cause antioxidative defense mechanisms in human osteoblasts via induction of •O2− and H2O2
Source: Sci Rep. 2017 Nov 6;7:14544. doi: 10.1038/s41598-017-14983-9 (PMC5673962; doi:10.1038/s41598-017-14983-9)
Supplement: Supplementary file 1 — Supplementary Figure 1 [file 41598_2017_14983_MOESM1_ESM.pdf]

Extremely low frequency pulsed electromagnetic fields cause antioxidative defense mechanisms in human osteoblasts via induction of $\bullet O_2^-$ and H_2O_2

Sabrina Ehnert,[1*] Anne-Kristin Fentz,[2] Anna Schreiner,[1] Johannes Birk,[1] Benjamin Wilbrand,[1] Patrick Ziegler,[1] Marie K. Reumann,[1] Hongbo Wang,[3] Karsten Falldorf,[2] Andreas K. Nussler[1]

Supplementary Figure 1

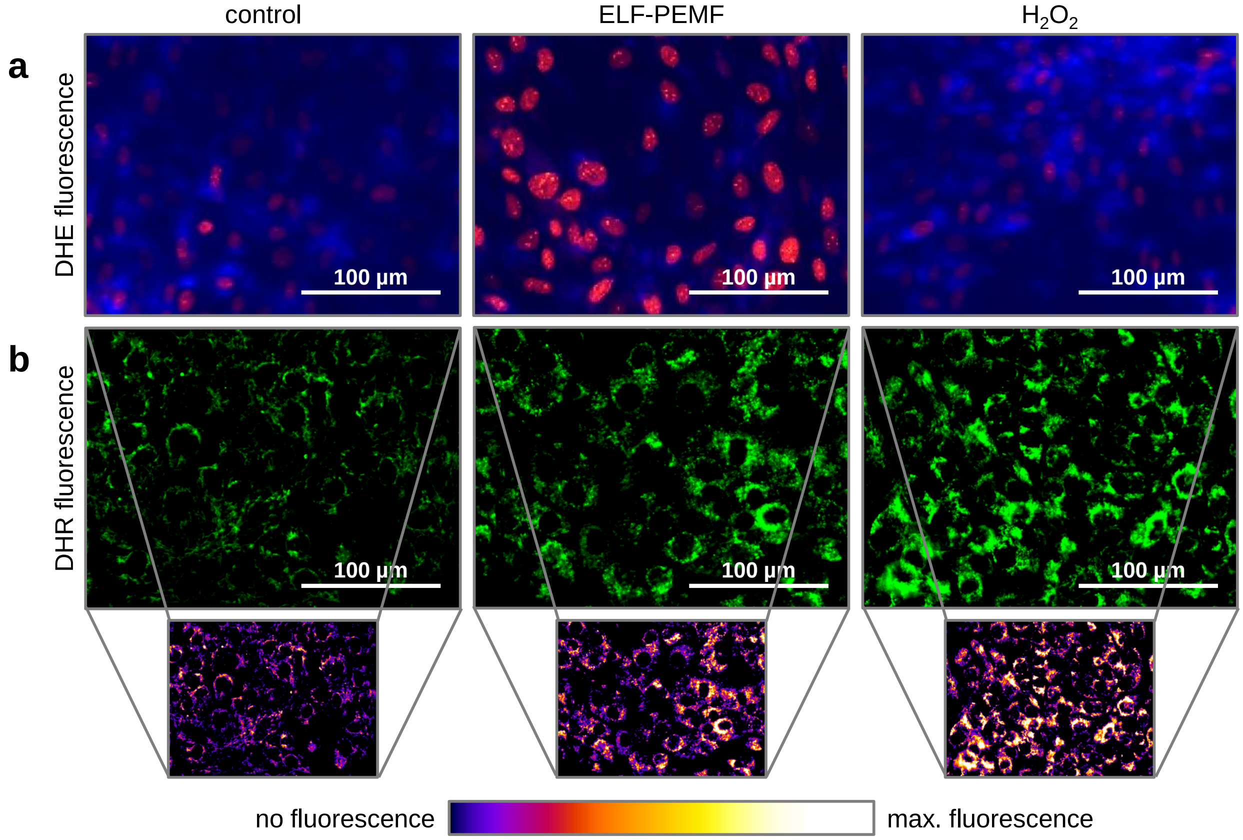

Supplementary Figure 1: Microscopic images of the DHE and DHR assay in hOBs. Directly after single exposure (7 min) to ELF-PEMF intracellular ROS levels were determined in hOBs using different fluorescent probes. (a) DHE assay was used to detect $\bullet O_2^-$. hOBs stimulated with 0.01% H_2O_2 were used as negative control to show specificity of the assays. (b) DHR123 assay was used to detect H_2O_2. In these cells stimulation with 0.01% H_2O_2 was used as positive control. Staining intensities were visualized by pseudo-color (fire) in ImageJ.
