# Supplementary material for: Extremely low frequency pulsed electromagnetic fields cause antioxidative defense mechanisms in human osteoblasts via induction of •O2− and H2O2
Source: Sci Rep. 2017 Nov 6;7:14544. doi: 10.1038/s41598-017-14983-9 (PMC5673962; doi:10.1038/s41598-017-14983-9)
Supplement: Supplementary file 2 — Supplementary Figure 2 [file 41598_2017_14983_MOESM2_ESM.pdf]

Extremely low frequency pulsed electromagnetic fields cause antioxidative defense mechanisms in human osteoblasts via induction of $\bullet O_2^-$ and H_2O_2

Sabrina Ehnert,[1*] Anne-Kristin Fentz,[2] Anna Schreiner,[1] Johannes Birk,[1] Benjamin Wilbrand,[1] Patrick Ziegler,[1] Marie K. Reumann,[1] Hongbo Wang,[3] Karsten Falldorf,[2] Andreas K. Nussler[1]

Supplementary Figure 2

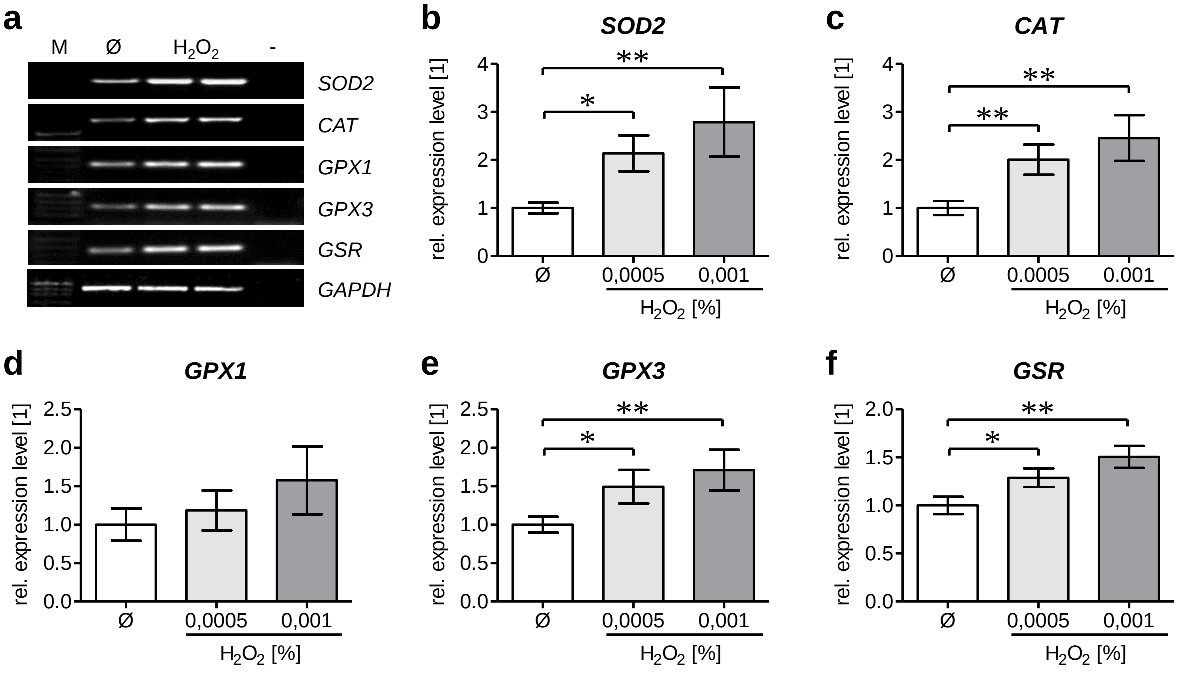

Supplementary Figure 2: H_2O_2 treatment increases expression of of antioxidative enzymes in hOBs. hOBs were osteogenically differentiated with or without 0.0005% or 0.001% H_2O_2. After 2 days mRNA levels of the antioxidative enzymes (*SOD2, CAT, GPX1, GPX3* and *GSR*) were determined by conventional RT-PCR. (a) Representative figure of the RT-PCR products for 1 donor. (b-f) Densitometric analysis (ImageJ software) was performed with individual samples (N = 3) analyzed twice (n = 2) to reduce small loading differences. The mean signal intensity of untreated cells was set as reference. * $p < 0.05$ and ** $p < 0.01$ as indicated (Kruskal-Wallis test followed by Dunn's multiple comparison test, $\alpha = 0.05$).
