# Supplementary material for: Extremely low frequency pulsed electromagnetic fields cause antioxidative defense mechanisms in human osteoblasts via induction of •O2− and H2O2
Source: Sci Rep. 2017 Nov 6;7:14544. doi: 10.1038/s41598-017-14983-9 (PMC5673962; doi:10.1038/s41598-017-14983-9)
Supplement: Supplementary file 3 — Supplementary Figure 3 [file 41598_2017_14983_MOESM3_ESM.pdf]

Extremely low frequency pulsed electromagnetic fields cause antioxidative defense mechanisms in human osteoblasts via induction of $\bullet O_2^-$ and H_2O_2

Sabrina Ehnert,[1*] Anne-Kristin Fentz,[2] Anna Schreiner,[1] Johannes Birk,[1] Benjamin Wilbrand,[1] Patrick Ziegler,[1] Marie K. Reumann,[1] Hongbo Wang,[3] Karsten Falldorf,[2] Andreas K. Nussler[1]

Supplementary Figure 3

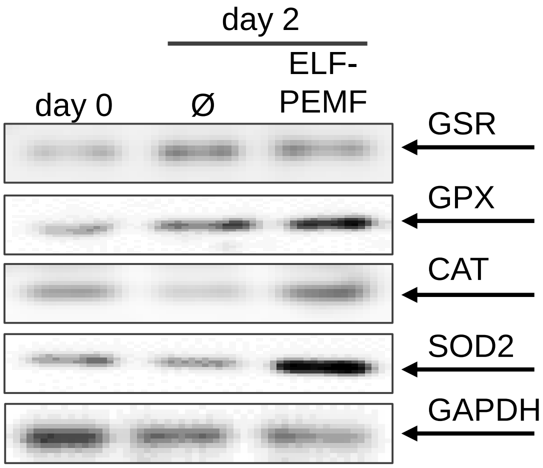

Supplementary Figure 3: ELF-PEMF exposure increases proteins levels of antioxidative enzymes in hOBs. hOBs were osteogenically differentiated with or without daily exposure to ELF-PEMF. After 2 days protein levels of SOD2, CAT, GPXs and GSR were determined by Western blot. Representative figure of Western blot signals for 1 donor.
